# What matters in digital health technology adoption for mental healthcare? - A multistakeholder qualitative study

**DOI:** 10.1177/20552076261469387

**Published:** 2026-08-10

**Authors:** Bo Wang, Ingunn Mundal, Karen L. Fortuna, Cecilie Katrine Utheim Grønvik, Trude Fløystad Eines, Marianne Storm

**Affiliations:** 1 Faculty of Health Sciences and Social Care, 5562 Molde University College, Molde, Norway; 2 Norwegian Centre for E-Health Research, University Hospital of North Norway, Tromsø, Norway; 3 Department of Community and Family Medicine, Geisel School of Medicine at Dartmouth, Hanover, NH, USA; 4 Department of Public Health, Faculty of Health Sciences, 56627 University of Stavanger, Stavanger, Norway

**Keywords:** digital mental health, mental health, technology, adoption, multistakeholder, qualitative study

## Abstract

**Objective:**

Digital health technologies hold great promises for supporting people with mental health challenges and promoting their well-being. However, various challenges persist in the adoption of technologies as care modalities in mental healthcare. The study aims to explore what matters in adopting technology in mental healthcare from a multistakeholders’ perspective.

**Methods:**

We conducted a qualitative interview study with 26 mental health stakeholders recruited from government authorities, regional health authorities/hospitals, municipalities, universities/research institutions, health industries/entrepreneurs/clusters, and user organizations. Semi-structured individual interviews were conducted via Microsoft Teams between August and October 2024. The interviews were audio recorded, transcribed into text, and analyzed using abductive thematic analysis.

**Results:**

Four main themes emerged from the analysis. Stakeholders identified flexible, personal adaptive technology supported by peer support and co-creation, coupled with continuous and tailored human follow-ups, as core values for people receiving mental healthcare. For successful adoption, stakeholders informed that such technology must be evidence-informed and demonstrate clear and scalable service delivery benefits in mental healthcare, while requiring visionary leadership and dedicated staff resources for organizational adoption. They further highlighted that nationwide access depends on compatible digital health systems and on clear policies and guidelines that support scaling for the benefit both service users and staff.

**Conclusion:**

Stakeholders considered technology adoption in mental healthcare as jointly shaped by human-centered values and broader contextual factors. They interpreted that effective strategies need to foster human connection while enabling broader access and knowledge transfer to ensure safe and scalable technology use.

## Background

Digital health technology holds great promises to promote health and well-being for people receiving mental healthcare.^1^ The COVID-19 pandemic has led to unprecedented adoption of digital health technology that has fundamentally altered the operations of mental healthcare.^2^ These technologies are delivered through various modalities (i.e., mobile applications, wearable sensors, patient portals, electronic health records, and social media), serving multiple purposes, such as treatment, symptom monitoring, and prevention.^3^ A large body of evidence has demonstrated promising results, including enhanced treatment outcomes and improved quality of life among service users in mental healthcare.^3–10^

Despite their huge potential, mental health services face several challenges in ensuring that the projected benefits of technology are applied in the area of greatest need.^11^ Staffing shortages among psychologists, community nurses, and social workers have limited services’ capacity to adopt technology to support people with mental health problems.^12^ In response to some of these challenges, recent years have witnessed a growing emphasis on recovery and incorporating user perspectives in mental healthcare to advocate for patient autonomy and account for the lived experiences of service users in mental healthcare, especially those with serious mental illness (SMI, such as schizophrenia, bipolar disorders, and major depressive disorders).^13^ Digital peer support has emerged as one important approach to implement this philosophy,^14^ with services empowered by technology and delivered by peer support workers who leverage their experiential knowledge of navigating mental health challenges to support others on their recovery journey.^14,15^ Studies demonstrate that such user-centered and recovery-based digital health technology represented an acceptable and feasible format that improves self-efficacy,^16,17^ hope,^16^ and various recovery-related outcomes^18^ in both peer support workers and service users.

While digital health technology becomes increasingly important in mental healthcare, service users in mental healthcare, including those with SMI, remain underrepresented in research evaluating these technologies.^19^ As new technologies such as artificial intelligence (AI) continue to revolutionize healthcare, there is an urgent call for a comprehensive, multifaceted understanding of the feasibility, utility, determinants, and normative foundations (i.e., how the technology is rooted in ethical, legal, and social principles to improve health outcomes while ensuring equity, safety, and human rights^20^) of digital health technology adoption in mental healthcare.^4,11^ By adoption, we refer to the process through which individuals, organizations, or systems learn about, accept, and integrate technologies into their routine practice, including their use, scale-up, spread, and sustainability across multiple stages and system levels. Understanding this complex phenomenon requires a broader perspective that includes the sociotechnical system in which the technology is situated. Defined by Shaw and Donia^21^(p. 1), a sociotechnical system is “the complex network of material devices, interpersonal relationships, organizational policies, corporate contracts, and government regulations that collectively shape how technologies are adopted and used”. A sociotechnical approach provides valuable insights into how individuals perceive, interpret, and utilize digital health technologies, ensuring they are accessible and have sustainable benefits for their users.^11,22^ It also examines how the adoption or non-adoption of these technologies influences the broader system, guiding meaningful and scalable technology implementation in mental healthcare.^11,21^

One such sociotechnical lens is Greenhalgh’s Nonadoption, Abandonment, Scale-up, Spread, and Sustainability (NASSS) framework.^23^ NASSS is based on the synthesis of earlier published technology innovation frameworks and relevant theories,^24^ such as the Diffusion of Innovation Theory^25^ and the Normalization Process Theory.^26^ The framework serves as a practical tool to evaluate challenges and complexity arising when introducing technology in health and social care services to understand and facilitate successful adoption.^23^ The NASSS framework includes seven domains, each of which can be analyzed in terms of its complexity level (e.g., intended and unintended consequences): condition; technology (features and material properties); value proposition; adopters (staff, patients, and carers); organization; the wider system; and the embedding and adaptation over time. The condition, technology, and adopters (domains 1, 2, and 4) focus on specific design aspects or technology use for particular conditions, comorbidities, or stakeholder groups. Meanwhile, the value, organization, and wider system (domains 3, 5, and 6) consider broader principles of whether a technology is worth introducing in the first place, organizational capabilities and readiness for adoption, and the simplicity or complexity of the wider institutional and sociocultural context of the technologies.^23,24^

Despite the growing use of a broad range of technologies in mental healthcare (such as mobile applications^27–29^), there is a need for more research that evaluates this complex sociotechnical phenomenon through a multi-stakeholder perspective.^11^ Such a perspective is crucial because stakeholders in mental healthcare, including clinicians, peer support workers, researchers, and policy makers, are often directly involved in various stages of technology adoption in the health system. They can bring multiple, complex and systematic perspectives on how technology can be useful, appropriate, and sustainable in practice. This study aims to explore the perspectives of stakeholders who have experience from various roles that are relevant for adopting technologies in mental healthcare. Hence, we seek to answer the following research question: what matters in digital health technology adoption in mental healthcare from a multistakeholder perspective? To achieve this, we applied the value, organization, and wider system domains of NASSS (Table 1), as they capture the value of technology for potential end-users, the organizational capacity and readiness for adoption, and the wider political, regulatory, and sociocultural context shaping technology use. This study forms a preparatory phase of a feasibility project testing a peer-delivered digital health technology for people with SMI.^28^ At this stage, we sought to develop a broader, multistakeholder-informed understanding of technology adoption in mental healthcare, so that potential facilitators and challenges could be identified to guide future safe and meaningful involvement of people with SMI in our sequent feasibility testing.

**Table 1.** The NASSS domains applied in this study.

NASSS domain	What it is	Complexity
Domain 3. Value proposition	Value for healthcare system, staff and service users	Difficulties in developing compelling cases for the technology or verifying assumptions regarding value generation^24^
Domain 5. Organization	Capacity, readiness, and extent of changes in adopting technology	The organizations’ capacity to embrace any service-level innovation, the readiness for specific technology, and the interdependence between organizations^24^
Domain 6. Wider system	Political context, regulatory issues, and sociocultural context	Negative perceptions of technology or specific barriers to its introduction from political or regulatory context^24^

NASSS framework - Nonadoption, Abandonment, Scale-up, Spread, and Sustainability framework

## Methods

### Study setting: The Norwegian healthcare system

The Norwegian healthcare system is funded primarily through general taxes and employer-employee payroll contributions, with all residents entitled to publicly funded healthcare services.^30^ The healthcare system operates at municipal and specialist levels. Municipalities provide primary care focusing on prevention, treatment, care, and daily assistance, including mental health services through general practitioners, emergency care, inpatient facilities, home healthcare, and supported housing.^31^ When municipal mental health services lack adequate competence and measures necessary for meeting the needs of service users (including those with SMI, based on symptoms, challenges, and impact on social and daily functioning), responsibility shifts to specialist mental health services, which provide inpatient and outpatient care through psychiatric hospitals and district mental health centers.^31,32^

Norway’s long geography and spread-out rural population, along with high education levels and widespread internet access, have made digital mental health services a focus for years.^32^ Digital psychotherapy, online consultations, and remote supervision are quickly developing and changing how mental healthcare is delivered.^32^ For example, many municipalities now use the *Assistant Self-Help* application for people with anxiety disorders.^33^ In specialist healthcare, the guided internet treatment program *eTreatment*, which has proven effective for anxiety and depression, has been rolled out across all four Norwegian health regions.^34^

### Study design, recruitment, and participants

Qualitative research is considered appropriate for exploring topics in depth from the perspectives of those involved, with interviews being particularly effective for elucidating people’s opinions and experiences.^35^ Therefore, we conducted a qualitative interview study to understand and navigate the landscape and nuances of adopting digital health technologies in mental healthcare. Individual interviews were conducted with multiple stakeholders that have experience from various roles that are relevant for adopting technologies in mental healthcare at different levels and settings of the Norwegian healthcare system. The involvement of different stakeholders provides us with a rich variety of perspectives and insights from different levels and standpoint within the healthcare system.^36^ Individual interviews encourage each stakeholder to share their perspectives on how to adopt digital health technologies in mental healthcare and their perceived impact, enabling us to gather and synthesize multiple viewpoints for a comprehensive understanding and explanation of the research question. The Consolidated criteria for reporting qualitative research (COREQ) checklist^37^ was consulted during the reporting of this qualitative interview study (Supplementary 1).

Eligible participants are those who: (1) are employed at healthcare organizations or institutions and (2) have work (i.e., development, adoption, or implementation) or user experience with digital health technology in mental healthcare (e.g., mobile apps, patient portals, remote consultations, digital peer support platforms and other modalities). The healthcare organizations or institutions include governmental authorities, regional health authorities, hospitals, municipalities, research institutions and universities, health care industries, entrepreneurs, innovation clusters and user organizations. Eligible participants could hold and have experience with various roles, including leaders or managers, advisors, researchers, health providers, peer support workers, and engineers.

We purposely sampled stakeholders who met the criteria at the above-mentioned healthcare organizations and institutions. Direct outreach via emails and telephone by the first author was used to inform them about the study and assess the eligibility of potential participants in their organizations or institutions. To avoid over-representation of any single viewpoint and ensure balanced participation across stakeholder groups, at least two participants were recruited from each of the above-mentioned healthcare organizations and institutions. Recruitment also considered the relevance of stakeholders’ experience with technology adoption in mental healthcare, as well as practical constraints on participation, such as availability, interest, and resources, particularly within government, the health industry, and user organizations. Analytic sufficiency was judged by whether each stakeholder group’s perspective was adequately reflected in the thematic structure, rather than by equal numerical representation. Each eligible participant received a participant information letter and participation consent (Supplementary 2) and was invited to consider participation. Those who expressed interest and provided written consent were enrolled in the study. Twenty-six eligible stakeholders were recruited for individual interviews with no dropouts. Table 2 describes demographic characteristics of the stakeholders.

**Table 2.** Demographic characteristics of our eligible stakeholders (n=26).

Characteristics		Stakeholder, n (%)
Age (y, n=26)	20-39	2 (8%)
	40-59	21 (80%)
	≥ 60	3 (12%)
Gender (n=26)	Female	13 (50%)
	Male	13 (50%)
Educational background in healthcare (n=26)^1^	Yes	14 (54%)
	No^2^	12 (46%)
Healthcare organization or institution (n=26)^3^	Governmental authority	2 (8%)
	Regional health authority/Hospital	6 (23%)
	Municipality	9 (34%)
	University/Research institution	6 (23%)
	Health industry/Entrepreneur/Cluster	3 (12%)
	User organization	2 (8%)^4^
Employment role of stakeholders (n=26)	Leader/manager	12 (46%)
	Employee	14 (54%)
Professional experience of stakeholders (y, n=26)	0 - 10	5 (20%)
	11 - 20	9 (35%)
	≥ 20	12 (46%)

^1^Educational background in healthcare includes psychiatry, psychology, psychiatric nursing, and social education.

^2^Non-healthcare educational backgrounds reported by the stakeholders include civil engineering, journalism and communication, pedagogy, social economics, health economics, and sociology.

^3^Two stakeholders are affiliated with both universities and hospitals, resulting in a total that exceeds 100%.

^4^Both stakeholders had lived experience of mental health conditions.

### Individual interview

The semi-structured, individual interview guide was developed by BW, MS, and IM, and revised by KLF, CKUG, and TFE. All authors refined and approved the final version of the interview guide. The interview guide was pilot tested internally with two colleagues, both with professional backgrounds in mental healthcare and experience with digital health technology, before being rolled out for participant interviews. The interview guide includes nine open-ended questions informed by *The Value Proposition, The Organization*, and *The Wider System* of the NASSS framework. The questions were adapted from the NASSS-CAT tools interview version^38^ and authors’ empirical knowledge within this field (see Table 3).

**Table 3.** Semi-structured interview guide.

Overview of questions​
• Tell me about yourself (age, gender, professional background, current positions, working years, experience with technology in mental healthcare).
The Value Proposition
• How do you assess the value of technology, such as mobile apps, for people with serious mental illness?
• How do you think the use of technology will affect the work routine of peer support workers (if applicable)?
• What impact do you think testing technology, such as mobile apps, will have on the organization?
The Organization
• What do you think are important resources/capacity of an organization in mental healthcare to adopt technology like mobile apps?
• What will it take for technology, like mobile apps, to be integrated into today’s workflows in mental healthcare?
• What do you think is important for successfully adopting technology into mental healthcare?
• What do you think could be the barriers to adopting technology, such as mobile apps, in mental healthcare?
The Wider System
• What external changes may affect adopting technology, such as mobile apps, over the next 3-5 years? How can it stay relevant to the future?

### Data collection

Individual interview data were collected via Microsoft Teams between August and October 2024. Each interview lasted between 30 to 60 minutes and was digitally recorded using a Dictaphone (a secure audio-recording platform used by Norwegian higher education institutions) and further imported into Nettskjema (a secured web-based survey tool that enables data collection used by Norwegian higher education institutions). BW conducted all 26 interviews, with either CKUG, TFE, or MS participating in each interview alongside BW. All interviews were conducted in Norwegian and transcribed to Norwegian text using the AI-assisted transcription tool in Nettskjema, which is an integrated tool in the Norwegian Agency for Shared Services in Education and Research system ensuring confidentiality and data protection. Each Norwegian transcription was quality-checked by at least two authors to verify words, expressions, and grammar.

### Data analysis

We conducted an abductive thematic analysis,^39^ a hybrid process that integrates inductive and deductive reasoning to iteratively explore and interpret the data. This approach allows us to generate and amplify logical explanations by intertwining empirical data with existing theoretical frameworks. The abductive thematic analysis followed the eight steps: (1) transcription and familiarization, (2) coding, (3) labeling of codes, (4) development of themes, (5) theorizing, (6) comparison of data sets, (7) data display, and (8) writing up the results.

In accordance with these steps, the authors first familiarized themselves with the transcribed interviews by performing narrow readings, listening to the recordings again to check for accuracy, and identifying meanings and issues that were of potential interest to the research question (step 1). Potential patterns within texts were then systematically coded through a reflectively inductive process, allowing openness to the data while minimizing the risks of overlooking expected results (step 2). To mitigate potential individual biases, at least two authors independently read and coded each transcript. Next, collaborative discussions were held to compare initial impressions and group semantically similar codes (step 3) into coherent clusters, forming preliminary themes (step 4). We determined that adequate themes had been reached when interview data, both within stakeholder groups as well as overall, no longer introduced new conceptual insights or meaningfully expanded the emerging themes.^40^ We deductively applied theories and concepts underpinning *the value propositions*, *the organization* and *the wider system* (domains 3, 5, and 6) of the NASSS framework and intertwined these with extracts from the transcribed data material for the interviews. Meanwhile, the authors inductively move back and forth between data and the NASSS framework to assess the extent to which existing theories, concepts (e.g., *coherence work* reflecting organizations’ capabilities for technology), and complexities described in NASSS can explain the data from the transcribed interviews (i.e., codes, preliminary themes, and the entire dataset) and to disclose any surprising aspects not covered by literature. Finally, the themes were reviewed and refined by assessing whether the codes within each theme coherently fit together and adequately represented the entire dataset. The themes and their names were collaboratively discussed and refined until the consensus was reached. The emergent themes were then theorized (step 5) to conceptualize meaningful interdependent relationships and possible connections among them, revealing how they collectively influence digital health technology adoption for mental healthcare.

Transcribed text was first imported to NVivo 14 for the analytical process and was later exported to Microsoft Excel to support collaborative discussion for the research group on emergent themes and theorization. The codes and the emerged themes during the data analysis process were then translated from Norwegian into English for the purpose of publication. All participant quotations were originally in Norwegian and were translated into English following a systematic multi-step procedure. The first author conducted the initial translation, focusing on semantic and contextual meaning. Translations were then reviewed and refined by co-authors to ensure accuracy, clarity and preservation of nuance. A brief audit approach was applied, where an independent researcher compared selected Norwegian quotations with their English versions and the associated categories. This process ensured consistency between original meaning, translation, and analytical interpretation.

### Author reflexivity

We recognized that our professional backgrounds and prior experiences could influence both the conduct of interviews and our interpretation of digital health technology adoption in mental healthcare. The interviews were conducted by authors trained in qualitative methods with professional backgrounds in health sciences and social care. To address potential power dynamics when interviewing stakeholders across multiple organizational levels, we emphasized neutrality, clarified the voluntary nature of participation, and encouraged open dialogue regardless of role or position. Throughout the analytical process, the authors adopted a reflective approach and collaborated closely.^41^ The six authors contributed diverse expertise, providing multiple perspectives on the data. We regularly discussed alternative explanations and reached a shared understanding that aligned with the study’s aim. This collaborative and reflective approach helped minimize individual bias and strengthened the credibility and trustworthiness of the findings.^42^

### Research ethics

The Norwegian Agency for Shared Services in Education and Research (SIKT) assessed the study (project nr. 269350) to ensure privacy measures and compliance with relevant regulations. The study followed the principles of the Helsinki Declaration.^43^ All participants took part voluntarily and were provided with information regarding confidentiality. They were informed that participation was voluntary and that they retained the right to withdraw from the study without consequences. Written informed consent was obtained from all participants before the individual interviews.

## Results

The results started with themes being presented in an ascending order from the ground level to the national level. The results conclude with the theorizing of emergent themes showcasing how they interconnect and interact within an ecosystem of technology adoption in mental healthcare. Table 4 illustrates the theme development process. A codebook detailing the audit trail of the results (step 3 of Thompson’s) is provided in Supplementary 3.

**Table 4.** Theme development process.

Example of data extracts	Code	Theme
*“The challenges are to create digital solutions that are tailored to the users’ needs and the individual preferences that the users have.”* (Stakeholder 23)	Personal adaptation	Personalized adaptive digital health technology through co-creation and peer support
*“The patient or user side should also be involved in the process, so that you get the [insight]that, they perceive the solution as something … that is better than what they’ve had before. Something can help them, but that it [digital solutions] can work.”* (Stakeholder 26)	Involving service users in the co-creation process	
	What is valuable for service users	
*“… we use a lot of peer support workers in our work with mental health service users, so they already have a very important role. There are many people within our municipality who have lived experience, and even more of them could be offered a type of job or positions that benefit users with lived experience than just special expertise”* (Stakeholder 24)	Peer support workers with lived experience are valuable resources	
*“Testing the solutions on a small scale before expanding them is very important, so that you have great credibility when presenting it as a solution that can be important for serious mental illness.”* (Stakeholder 11)	Technology needs to be tested before implementation	Evidence-informed organizational adoption for digital mental healthcare
*“If there’s very little reward for organizations adopting new technology, then they will not be adopted. This is an important point here - funding controls everything.”* (Stakeholder 2)	Organizations need incentives for adoption	
*“I believe it is extremely important that when using digital solutions, they must be rooted in the sectors that can be professionally managed, and leaders who should be responsible for the management process.”* (Stakeholder 13)	Leadership is vital	
*“You must have dedicated staff, meaning that having someone hired to implement or use a solution. For example, if you hire psychologists to run e-health and that’ll be their job, that’s kind of the instruction for them”* (Stakeholder 6)	Dedicated personnel facilitate adoption	
*“There are no good system means that we constantly have to create our own systems to take care of the use in system we have, so there’s no connection in patient information flow or exchange.”* (Stakeholder 20)	Needs integrated and well-functioning digital health system	A well-integrated and sustainable digital health system to support mental healthcare
*“… the problem is that there are so many different platforms being established and they don’t communicate well to support serious mental illness, there should be a national health platform with all technologies and agencies in there and it’s secure”* (Stakeholder 24)	Compatible platform granting safe access to various technologies	
*“There are a number of technical aspects here that make these digital tools work, so that there will be a need for a type of operational support along the way.”* (Stakeholder 13)	Consistent operational support	
*“Nationally, we must implement or have an endorsement of the EU regulations, so that we facilitate the use of solutions developed in Europe. That means that one agrees on a set of requirements that are the same for Norway, Sweden, France, Germany, etc. There are requirements for safety, for assessing treatment effect and side effects and so on.”* (Stakeholder 9)	Follow European Union and national regulations	Clear and enforced national regulatory frameworks and strategies
*“We always look at national guidelines when we are going to develop services, because it’s easier to get it introduced locally when we get some guidelines with resources saying that we should actually do it.”* (Stakeholder 18)	National initiatives with resources	
*“We are under pressure and many people have to save money … The biggest barrier is finances and silo funding in municipalities.”* (Stakeholder 18)	Pressure on funding structure	
*“I think that perhaps national authorities need to make some procurements, possibly approve technologies, so that it becomes easier for municipalities and specialist health services to get started and use the tools that you are developing. Because once you have developed it, it is not automatic that it will spread like wildfire. Many municipalities are small, they do not have knowledge about procurement.”* (Stakeholder 9)	Clear procurement process helps adoption	

### Theme 1: Personalized adaptive digital health technology through co-creation and peer support

Some stakeholders emphasized that for people receiving support from mental healthcare, the primary value of digital health technology should be first and foremost rooted in flexibility and personal adaptation to accommodate a holistic view of individuals’ needs, choices, and preferences. Regular, continuous follow-up emerged as an irreplaceable component for supporting this personalized adaptive approach, as one highlighted:

*“I think follow-up and feedback are absolutely crucial for them, because otherwise, they might open the app once or twice and then stop using it. The digital solution needs to engage users, making sure they like it and can easily provide feedback. The most important thing is regular follow-up. Before the next meeting, it’s important to review what has happened and what they’ve been working on, so you can say, ‘Yes, I see what you’ve done,’ right?”* (Stakeholder 1, governmental authority)

Several accounts pointed to co-creation with end-users (i.e., service users and health providers) from the early developmental stages as a prerequisite for ensuring flexibility and use of digital health technology. Some described the process of co-creation as a responsible “*bi-directional dialog*” or a “*joint choice*”, allowing room for a shared understanding of the technology’s value for end-users rather than relying solely on the experiences of health providers or entrepreneurial perspectives. One participant highlighted that co-creation is particularly important when addressing potential discrepancies between service providers’ perceptions of users’ needs and the users’ actual needs:

*“My experience tells me that there is often a big difference between what therapists – and perhaps also managers – believe patients want and what patients actually want when you ask them. We experienced this with video consultations, where I perceived that there was a much more conservative attitude from the treatment level than from the patients themselves. We probably need to listen more closely to the patients when adapting digital health technology.”* (Stakeholder 11, hospital)

Peer support workers with lived experience were highlighted by diverse stakeholders (clinicians, leaders, project managers, and former peer support workers) as invaluable resources and a *“driving force”* for achieving meaningful co-creation and ensuring digital health technology well-captures the identities, choices, and preferences of people with mental health problems. Some saw the advantage of peer support workers stemming from their less constrained roles compared to traditional health professionals, which allowed them to connect with vulnerable and hard-to-reach individuals through a non-hierarchical, culturally equitable approach. One said:

*“I think they can play an even bigger role in digital mental healthcare for serious mental illness! Since they do not have a very defined role, it may be easier to implement such platforms among them, and then we could utilize lived experience beyond just specialized expertise, so maybe more people could benefit.”* (Stakeholder 22, municipality)

As regulations for mental health peer support continue to evolve, some viewed this transitional phase as providing greater opportunities to integrate new technologies among peer support workers. They see this transition as enabling peer support workers to challenge the social stigma, especially for those with SMI, and support service users’ social inclusion by bridging traditional and digital mental healthcare. As one former peer support worker noted:

*“We should stay connected to technology and innovation in our field and learn to do our jobs in a new way, because now we have the opportunity to challenge the dangerous idea of ‘once an addict, always an addict’ and encourage our users to be part of the society, at least to some extent.”* (Stakeholder 7, regional health authority)

Some stakeholders from municipalities and hospitals emphasized that clear responsibilities, roles, and work boundaries must be established in advance for peer support workers to effectively manage service users’ emotional situations (such as anger or despair) and provide appropriate follow-up care for individuals. They stressed that these boundaries are essential for protecting peer support workers’ own health and well-being, as some peers may be vulnerable to triggering certain situations when supporting people and their recovery. One preventive approach highlighted by one from the municipality was the use of role-playing to clarify roles and responsibilities:

*“There are situations that you must address in advance that can be difficult to handle when emotions run high. If someone sends a cry for help at 2 AM to a peer support worker, how do we take care of both the patient and the peer support worker? Where are the boundaries? In our municipality, we have regular role-playing to discuss different experiences, responsibilities, and roles.”* (Stakeholder 22, municipality)

Several stakeholders from government, municipalities, regional health authorities, and user organizations expressed concern that some may be unwilling or unable to use digital health technologies due to concentration problems, paranoia symptoms, limited digital literacy, lack of Internet access, infrastructure, or equipment (e.g., most secure logins request digital bank ID in Norway, but some users do not have one), which affects (or worsens) their digital engagement. They noted that addressing these challenges can be complicated by the diverse demographic characteristics, including variations in age, socioeconomic status, and functional abilities. While some emphasized the importance of respecting users’ choices, others highlighted the importance of providing easily accessible, human-centered training and guidance for both service users and their peer support workers. A stakeholder from a user organization, suggested training can build users’ confidence and motivation for using technology, upskilling digital competencies, and prevent avoidable digital exclusion:

*“Easily accessible training, guidance, and good support are important for those who are actually going to use it in their recovery. It takes some time to catch up with those who find it difficult because those who struggle don’t talk about it out loud*. *They might keep their mouth shut and just pretend it’s nothing and stay quiet when it’s talked about. You have to be careful that it doesn’t be so controlling and uncomfortable*.*”* (Stakeholder 25, user organization)

### Theme 2: Evidence-informed organizational adoption for digital mental healthcare

Many participants talked about the organizational value of digital health technology in mental healthcare in terms of increased capacity and productivity (for example, reducing waiting lists), alongside reduced costs and resource requirements, which means that adopting technology was perceived as both operationally beneficial and financially justifiable. However, they further emphasized that adoption decisions were primarily based on evidence demonstrating the feasibility and good effects of these technologies. They argued that testing conducted both during the pilot phases and during broader adaptation is essential for credibility, transparency, and systematic learning about how specific solutions benefit service users, like one said:

*“We have many examples of digital solutions, like the recent Helseplattformen [Health platform, an electronic health records system from the US] in the Middle Norway, being forced into services or hospitals without actually being sufficiently tested beforehand over the past few years. This often creates more trouble than the solution itself, wasting money on things that don’t work instead of on those with serious or concurrent illnesses who fall between systems.”* (Stakeholder 7, regional health authority)

Several stakeholders emphasized that technology that has been successfully adopted by organizations (such as *Assistant Self-Help* app in multiple Norwegian municipalities) demonstrated tangible operational benefits or economic gains and incentives for healthcare services. One stakeholder from a municipality advocated that specific technology is needed to prove how it can increase service providers’ work capacity:

*“We must demonstrate that this investment yields cost savings somewhere, like reducing waiting lists. If not, we'll just be told to use something else that can achieve the same effect, or to work differently. If we can't show these investments have an impact, we'll be asked to try other tools that cost less but deliver the same results.”* (Stakeholder 16, municipality)

Leadership was described by many stakeholders from hospitals, universities, and research institutions, and municipalities as the “*Alpha and Omega*” for organizational adoption of technology in mental healthcare. Many viewed effective leadership as a two-sided managerial responsibility (i.e., both leaders themselves and employees). Several stakeholders emphasized that leaders must *“be willing to get on board”* themselves, recognizing the value of digital health technologies in mental healthcare to foster a collaborative culture towards adoption. They noted that it requires leaders to have a good understanding of new technologies, the ability to communicate clearly and effectively, and the skill to anchor the value and goals with employees. Many stakeholders described effective leadership as balancing decisive governance with inclusive management approaches that integrate valuable employee insights into implementation decisions, as one of them explained:

*“The leadership can't be bottom-up, it has to be top-down. There are many processes and hidden expenses involving procurement, administration, training, and new work routines, so you need the right infrastructure and a shift to a new way of working. You have to secure professional buy-ins from your employees, and their voices must be heard. There will be some resistance, but if you want to realize the benefits, you must engage and collaborate with the people within your organization as a team.”* (Stakeholder 9, governmental authority)

When resistance arises toward adopting new technologies in mental healthcare (usually due to changing work routines or workload), some stakeholders emphasized the importance of onboarding dedicated personnel and human expertise to support the transition process. Beyond traditional IT consultants or technicians, several described emerging specialist roles focused on innovation, digitalization, implementation, or a combination of these skills. Positions like *“digitalization consultants”* or *“innovation advisors”* were identified by several stakeholders as new specialists in some hospitals and municipalities who facilitated the integration of technology in mental healthcare. One highlighted:

*“What I heard is that xxx Hospital employed an innovation advisor who had a mandate to streamline the departments digital operations. It felt like they'd been able to put a lot of new solutions in place.”* (Stakeholder 4, health industry)

### Theme 3: A well-integrated and sustainable digital health system to support mental healthcare

Generally, our stakeholders reflected on the importance of new technologies being compatible with existing digital health systems and infrastructure and fitting within health providers’ workflows to support efficiency and accuracy in mental healthcare. However, some from municipalities and universities quickly pointed out the practical challenges of achieving such integration. They noted that Norwegian hospitals and municipalities rely on fragmented, non-standardized systems (like the existing electronic health records systems), which demand complicated (and usually costly) technical adjustments and changes to daily work routines and can further hinder access to digital health technologies in mental healthcare. While the public healthcare services remain too rigid for the quick implementation of such adaptations, many expressed concerns that these challenges could, in turn, disrupt the continuity of mental healthcare. One remarked:

*“Everyone is equally dissatisfied with these digital health systems because they don’t talk to each other either. If you want your solution to work for different people with serious mental illness, it shouldn’t be required to be integrated into the current digital health systems, nor too much change in the way we work, or it becomes difficult to follow up on these patients in a busy everyday life*.*”* (Stakeholder 20, municipality)

Adding to this challenge, several stakeholders worried that limited budgets and the unpredictable expenses required to integrate technologies within fragmented digital health systems present major constraints to sustaining these technologies in mental healthcare. One rhetorically described the financial burden as *“the sky is the limit”* and said:

*“We have already invested a lot in equipment to get started and bought and rebuilt machines to handle and process everything*. *I think for many other organizations and municipalities, especially small ones, it’s a lot of expenses and pressure that we might not be able to bear.”* (Stakeholder 24, municipality)

Several stakeholders across government, hospitals, universities, health industries, and municipalities expressed a preference for national, compatible, standardized platforms accessible to healthcare organizations, viewing it as pathway towards more equal, sustainable, and cost-effective solutions. Given the heterogeneity of technology, one suggested that healthcare system could take inspiration from Spotify (a music app):

*“I think we need a sort of ‘Spotify-model’ within Helsenorge.no [the Norwegian national health platform] to put different solutions and software on. It’ll be a common platform that you can log in to and find different offers not just for therapists, but also for users with SMI when they have a lot of different digital solutions that they need to access. We hope that the health authorities have thought the same way too, especially since we’ve had a lot of different systems that were supposed to handle different processes.”* (Stakeholder 11, hospital)

### Theme 4: Clear and enforced national regulatory frameworks and strategies

Broadly speaking, our stakeholders perceived that technology adoption in mental healthcare requires compliance with multiple regulatory frameworks and strategies. These include, for example, national and regional legislation (including locally enforced health and fiscal policies), information safety and data privacy protocols (such as Data Protection Officers in research), and European Union regulations (like the General Data Protection Regulation, GDPR) – as the participants consistently emphasized. Building on these regulatory requirements, many also highlighted national initiatives with clear implementation resources and guidelines as the essential foundation for embedding technologies in mental healthcare, noting that *“it has to be invested simply from the highest levels nationally.”* Some specifically pointed to a self-management mobile app (*Assistant Self-Help*) as an example that gained adoption in many Norwegian municipalities due to its clear national backing with resources. Without such structured strategies, problems quickly emerge, as one explained:

*“When something went wrong and there were no clear guidelines for it, we didn’t know where to turn for help, which means our service costs became quickly expensive. We need all these procedures and routines related to the implementation and how to conduct different technologies among different groups of patients in mental healthcare.”* (Stakeholder 19, municipality)

Several stakeholders from hospitals, municipalities, and research institutions described the persistent challenges with the fragmented funding structure in the Norwegian public mental healthcare, where budgets were siloed across sectors and level of governance, which further limited the ability to coordinate and adopted technology that could benefit the entire healthcare system:

*“… to get innovation introduced, it almost has to save money for whoever implements it. The municipality will ask why they should pay for hospital savings, and hospitals question paying for municipal savings. The financing is so fragmented that the public sector should better see the whole picture — it's all the same money!”* (Stakeholder 2, hospital/research institution)

Some stakeholders from universities and municipalities further pointed out the importance of establishing a national, professional and clear procurement process to support mental healthcare organizations and institutions adopting digital health technology. They stressed that licensing agreements, payment responsibilities, access duration, and potential user fees must be clearly defined, particularly for small municipalities with limited procurement expertise or knowledge. One stakeholder emphasized that a standardized procurement process could enable hospitals or municipalities to access effective technologies:

*“If there are licensed programs, it must be clarified who pays for the license. Sometimes it's the Directorate of Health that provides national access, while other times it can be health enterprises or municipalities. This needs to be clarified because many small municipalities don't have any knowledge about procurement. That process has to be in place before the tool can be used.”* (Stakeholder 13, university)

### Theorizing emergent themes

**Figure 1.**
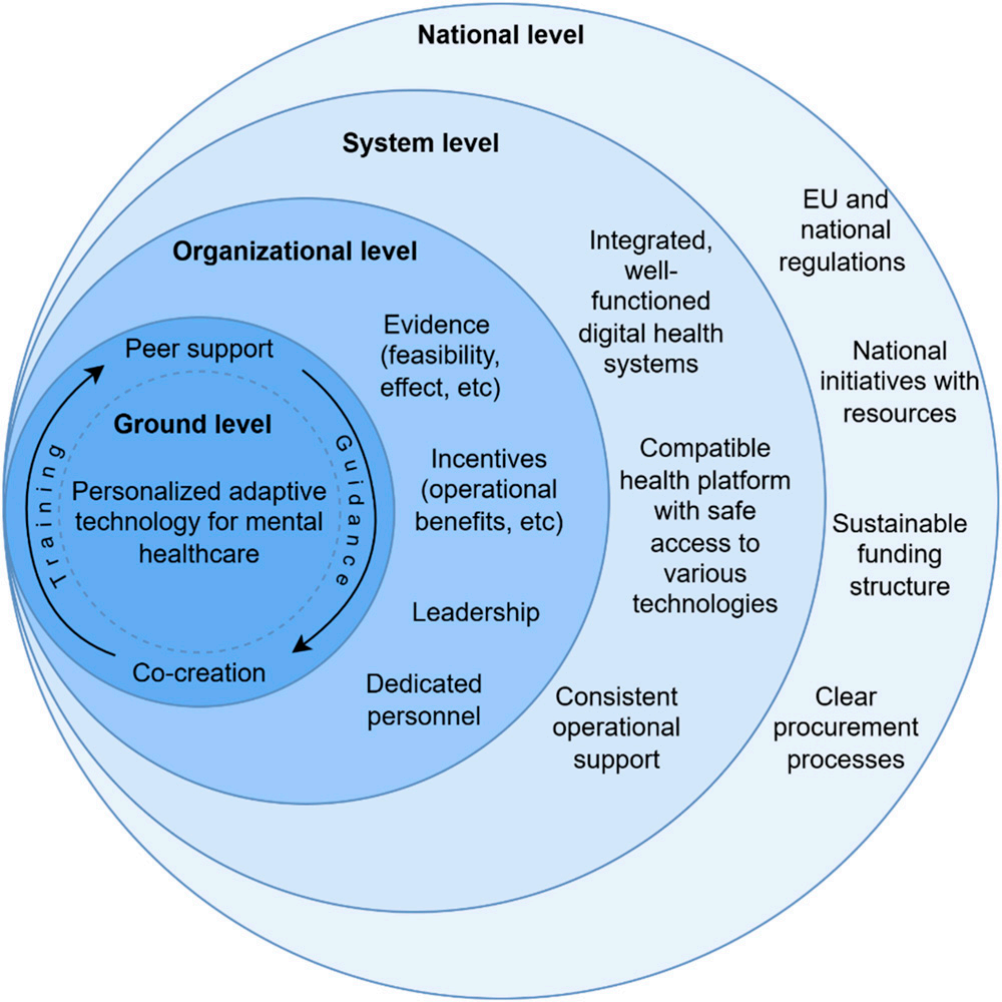
Theorizing emergent themes.

Following Thompson’s method of abductive thematic analysis at the data display stage (step 7), we theorized the emergent themes to conceptualize the interdependence between themes (Figure 1). Theme 1 relates to *the value proposition* domain of NASSS, by illustrating how human factors facilitate ground level adoption and relates to the demands in demonstrating a technology’s value. Themes 2 and 3 connect to *the organization* domain of NASSS by showing how leadership, physical resources, and their continuous two-way communication influence organizational readiness to adopt technologies that support people receiving mental healthcare (organization and system level). Theme 4 connects to *the wider system* of NASSS by highlighting how legal and regulatory conditions significantly shape development and implementation of technologies in mental healthcare (national level). As Figure 1 illustrates, flexible, personal adaptive technology sits at the heart of digital health technology adoption in mental healthcare, with all surrounding activities serving this central purpose. Peer support and co-creation form a reciprocal feedback loop through lived experience exchanges, directly supporting this person-centered purpose. Accessible training and consistent guidance for end-users (i.e. service users, peer support workers, health professionals) should be maintained to support meaningful and sustainable engagement with technology. Organizational support translates individual needs into workable strategies, while system-level infrastructure and national frameworks provide the necessary structure and standards, all oriented toward the central purpose of supporting people with mental health challenges through right-based, recovery-oriented approach. This creates an ecosystem where insights flow upward from individuals with lived experience while purpose-led resources and strategies flow downward, continuously and reflectively evolving to truly serve this population.

## Discussion

We identified four main themes explaining what matters in digital health technology adoption in mental healthcare from multistakeholders’ perspectives. Stakeholders perceived that a flexible, personalized adaptive solution that incorporates lived experience through peer support and co-creation, with well-timed training for service users and providers along the way that respectfully reflects end-users’ needs and preferences, as the core values when adopting technology in mental healthcare. Such values are embedded within a comprehensive framework that emphasizes the importance of feasible, effective, and cost-efficient technology to facilitate organizational adoption. Our stakeholders believe that to achieve this, purpose-led resources coupled with visionary leadership are essential for facilitating the decision-making process. They further emphasized that clear policies and well-designed digital health systems can enable organizations to move successfully from initial adoption to upscaling solutions into mainstreamed services that benefit both people receiving mental healthcare and staff involved.

Our findings showed that mental health stakeholders view personally adaptive digital technology, supported by accessible human support, as critically important in mental healthcare, including for those with SMI. The findings are consistent with decades of evidence demonstrating the vital role of face-to-face interaction in achieving effective digital solutions for people with mental health problems.^44–46^ However, we observed digital exclusion may occur among people receiving mental healthcare, particularly those with SMI, due to factors such as concentration problems, paranoia, or advanced age. This finding echoes with Spanakis et al.,^47^ and we consider that it may potentially exacerbate social isolation and hinder community integration. NASSS claimed that the complexity in the value proposition of a technology is associated with limited adoption.^23^ In our study, the findings suggest that limited adoption, particularly among people with SMI, may better be understood within the diverse social, cultural, cognitive, and environmental contexts that contribute to digital exclusion. One critical strategy can be partnering with this population to co-design digital health technologies that capture personalized preferences and unrecognized concerns early in the developmental phase.^48^ Integrating human factors enhances the partnering process to facilitate engagement among vulnerable populations who commonly disengage from digital health technology use.^48^ Another potential strategy can be upskilling digital health literacy (i.e., the ability to find and understand health-related information online and apply this knowledge to self-manage conditions^47^) for those living with chronic and complex mental health challenges,^47^ for example, to empower users’ ability and motivation to self-manage important administrative tasks including making online appointments and handling repeated medication prescriptions.^11,47^ Furthermore, it may be beneficial to ensure transparent disclosure in advance of technology use and functions, coupled with clear options for people to “opt out” in favor of in-person care,^49^ in order to safeguard long-term trust and reliability.

We found that peer support workers were perceived by several stakeholders across regional health authorities, hospitals, municipalities, and user organizations as valuable resources in facilitating flexible, person-centered technologies in mental healthcare and playing an increasingly important role in shaping rights-based and recovery-oriented technology use, particularly for those with SMI. This finding is supported by several studies of Fortuna et al.^14,50,51^ which support that peer support may foster self-management and digital health engagement. We interpret peer support workers’ impact as their “two-eye seeing” perspectives. The user perspective enables them to reach individuals with a wide range of lived experiences (including those “hard-to-reach”), empathetically recognize the bumpy road of recovery, and ensure individuals’ autonomy, dignity, and choices are integrated and reflected into digital adoption.^14^ In contrast, their staff perspective helps organizations navigate ethical complexities to respond appropriately to changing needs for people receiving mental healthcare. A concern arising from the “two-eye seeing” perspectives is how to clarify boundaries when peer support workers incorporate their lived experience into technology use.^52^ As mental health peer support rapidly grows in Norway and other Western societies,^53^ our findings recognize the importance of establishing clear responsibilities and boundaries for peer support workers in technology adoption to protect their own health and wellbeing. Our findings indicated that simulation-based training could be a practical training approach to prepare them to engage with technology that supports people receiving mental healthcare. To our knowledge, the role of peer support in mitigating or preventing digital exclusion in the SMI context has not yet been explicitly addressed within the NASSS framework, suggesting an opportunity to incorporate this new perspective in *the value proposition* when evaluating digital mental health projects for people receiving mental healthcare.

A common challenge we noticed from most stakeholders across different levels of mental healthcare is the difficulties in integrating technology into mental healthcare into compatible working systems and routines in healthcare organizations, which often complicates providers’ practice and disrupt continuity of care. This challenge aligns with the complexity described in *the organization* of NASSS^23^ and echoes previous studies on technology adoption challenges in mental healthcare settings.^1,54^ Our results indicated that strong leadership and facilitating resources for digitalization and implementation may support coordination and help address integration challenges in mental healthcare, as also observed by Eyre et al.^55^ However, we notice that the cultural adaptation of technologies (meaning the extent to which new technologies should be “tamed” and fine-grained for local contextual settings^56^) warrants attention throughout the development and deployment processes. Our results suggest that this may be particularly important for Norway and countries with smaller populations, where there may be a greater need to domesticate foreign mental health-related innovations to develop a shared understanding of local ethical complexities to improve the scalability of effective technologies.^57^ One example of such practice is the DigiPer digital health intervention for people with SMI.^58,59^ Upon translation and introduction to Norway, researchers tested its usability and desirability, and co-created recovery-oriented training modules with service users, peer support workers, and mental health professionals to capture cultural priorities for local end-users.^59^ Our research group continues to explore its implementation in Norwegian mental health services through the lens of *the condition*, *the technology*, and *the adopters* of NASSS, and using it as a case example to inform future culturally adaptive technologies for recovery-oriented mental healthcare for people with SMI.

Across all stakeholder groups, a persistent concern was that financial burdens coupled with unclear guidance on technology adoption in mental healthcare represent major barriers in organizations (particularly in municipalities) and the systems, aligning with the complexities described in *the organization* and *the wider system* of NASSS.^23^ Despite the Norwegian government’s promotion of the use of technology in mental healthcare, budget constraints and resource gaps, including a lack of or absence of peer support workers, remain significant challenges across municipalities. To potentially inform more targeted national funding streams for personalized technology in mental healthcare, our results suggested that the existing evidence base for effective and cost-effective technologies could be expanded through multisectoral, follow-up research (beyond piloting) that explores scalable benefits relevant to the evolving needs of service users and providers. At the same time, sectors such as municipalities, with more experience in technology adoption may serve as champions by sharing knowledge with less experienced organizations, creating further opportunities to support adoption.

### Strengths and limitations

The key strength of this study is the involvement of a broad variation of stakeholders across different levels and settings of mental healthcare. Each stakeholder brings diverse angles and perspectives on technology adoption in mental healthcare, enabling us to develop a comprehensive, multifaceted understanding of this dynamic and complex phenomenon. Over half of the stakeholders have healthcare backgrounds with direct professional experience working within mental healthcare, including SMI. Two stakeholders from user organizations have lived experienced mental health conditions. The inclusion of two stakeholders with lived experience of mental health challenges strengthens the credibility of our findings. However, the limited numbers of such participants may have limited the range of lived-experience perspectives represented. The involvement of multiple researchers from varied professional backgrounds and perspectives in the analysis process constitutes an additional strength that enhances the trustworthiness of this study.

Certain limitations should be considered when interpreting our results. First, a subset of participants showed lower engagement during the conversations and required direct prompt to articulate their perspectives. This may have been due to varying experiences from mental healthcare, and stakeholders having limited experiences regarding the use of technology in mental healthcare - making some more conservative and others more open to new possibilities – or to the online interview format, which may have affected rapport, disclosure, and the depth of responses for certain participants. Second, some findings may be specific to Norwegian and Nordic contexts, potentially influenced by public-funded healthcare systems, cultural factors such as flat organizational structures that emphasize employee voice, and advanced digital infrastructure with widespread internet access. Finally, despite the rigorous analytical approach, the interpretation of qualitative data inevitably involves subjective experience and judgment, which may have influenced our understanding and presentation of the findings. Future research could address this limitation through methodological triangulation.

## Conclusions

Adopting digital health technologies in mental healthcare requires a sociotechnical lens to understand the complex phenomena across multiple stakeholder levels. Our main contribution is to show that stakeholders view flexible, personally adapted technology supported by peer support and co-creation as core values in mental healthcare. These values appear to interact reciprocally with, and are influenced by, purpose-aligned actors and resources across organizations, digital health systems, and the national level. Stakeholders viewed that regular in-person follow-ups, supported by accessible training and guidance, are important components of implementing personalized technology in mental healthcare. Stakeholders also recognized effective leadership and dedicated financial and human resources facilitate technology needed to be integrated into existing systems and routines, while clear policy strategies and working guidelines support transition from successful pilot projects to mainstream mental healthcare. Experiences and knowledge from clinicians, researchers, leaders, and organizations may iteratively inform government adaptation of guidelines that ensure person-centered, right-based, and recovery-oriented digital health technology in mental healthcare.

## Supplemental material

Supplemental material - What matters in digital health technology adoption for mental healthcare? - A multistakeholder qualitative study Supplemental material for What matters in digital health technology adoption for mental healthcare? - A multistakeholder qualitative study by Bo Wang, Ingunn Mundal, Karen L. Fortuna, Cecilie Katrine Utheim Grønvik, Trude Fløystad Eines, Marianne Storm in DIGITAL HEALTH.

Supplemental material - What matters in digital health technology adoption for mental healthcare? - A multistakeholder qualitative study Supplemental material for What matters in digital health technology adoption for mental healthcare? - A multistakeholder qualitative study by Bo Wang, Ingunn Mundal, Karen L Fortuna, Cecilie Katrine Utheim Grønvik, Trude Fløystad Eines, Marianne Storm in DIGITAL HEALTH.

Supplemental material - What matters in digital health technology adoption for mental healthcare? - A multistakeholder qualitative study Supplemental material for What matters in digital health technology adoption for mental healthcare? - A multistakeholder qualitative study by Bo Wang, Ingunn Mundal, Karen L. Fortuna, Cecilie Katrine Utheim Grønvik, Trude Fløystad Eines, Marianne Storm in DIGITAL HEALTH.

## Data Availability

Data are available to appropriate academic parties upon reasonable requests to the corresponding author.*

## Appendix

### List of abbreviations

AI: Artificial intelligence
NASSS: Nonadoption, Abandonment, Scale-up, Spread, and Sustainability
SMI: Serious mental illness.
