# Supplementary material for: What matters in digital health technology adoption for mental healthcare? - A multistakeholder qualitative study
Source: Digit Health. 2026 Aug 10;12:20552076261469387. doi: 10.1177/20552076261469387 (PMC13458126; doi:10.1177/20552076261469387)
Supplement: Supplementary file 2 [file sj-pdf-2-dhj-10.1177_20552076261469387.pdf]

Would you like to participate in an interview for the research project - "PeerTECH" digital peer support for people with mental illness

This is a question for those with experience with **digital health** (mobile apps, wearables, web-based programs, telemedicine, etc.) in **the field of mental health/mental health work** to participate in a PhD project where the purpose is to test a Norwegian version of an American application (app), PeerTECH. In this letter, we provide you with information about the goals of the project and what participation in the first sub-study will entail for you.

Purpose
Peer support is a form of social support that utilizes the practical knowledge and ability to see things in context, which comes from having lived with the experience of having a mental illness, i.e. personal experience. Peer support is based on a philosophy that promotes self-determination, personal empowerment and freedom of choice where personal experience is the basis for organized support. There are several ways to organize peer activities. At Dartmouth College in the United States, researchers have developed an app, PeerTECH, to help adults deal with both mental and somatic illness. The app must be used in collaboration with a peer. It includes various training modules with information and guided support for self-care. We have translated and adapted the app to the Norwegian context and the Norwegian health service. In this interview study, we will gather knowledge about factors that may affect the use of a peer-supported app such as PeerTECH in Norway.

Who is responsible for the research project?
Molde University College is responsible for the project. The project is led by Professor Marianne Storm. Bo Wang is a PhD candidate in the project. The project is carried out in collaboration with Professor Ingunn Mundal (HiMolde), Assistant Professor Karen Fortuna (Dartmouth College, USA), Associate Professor Trude Eines (HiMolde), and Associate Professor Cecilie Grønvik (HiMolde).

Why are you being asked to participate?
We are looking for someone who has experience in the field of mental health, knowledge of digital health and peer support. We will interview 15-20 people with relevant experience from the Directorate of Health, health trusts, municipalities, businesses, user organizations and universities or colleges. To recruit participants, we will use the snowball method and contact managers in the relevant unit who ask relevant employees if they want to participate in an interview. We will also contact user organisations such as the Centre for Experience and Mental Health to provide information about the study.

What does it mean for you to participate? In this project, we invite you to attend an individual interview. The interview will be conducted during the autumn of 2024. In the interview, we want to gain insight into your experiences and perspectives on the use and introduction of digital health in the mental health field. The interview will take place via Microsoft Teams or in person. The duration of the interview is approx. 1 hour. Sound recordings and transcribed material must be used.

Participation in the project is voluntary. If you choose to participate, you may withdraw your consent at any time without giving any reason. All information about you will be deleted. There will be no negative consequences for you if you do not want to participate or choose to withdraw.

Your privacy – how we store and use your information.

We will only use your information for the purposes we have described in this writing. We treat the information confidentially and in accordance with the data protection regulations.

- The interview guide is designed so that you can comment on the various topics without hindrance from confidentiality.
- We will record the call with an audio recorder, which will then be transcribed into text.
- A code links your name to your voice from the audio file through a list of names.
- The list of names will be separated from text and audio files and kept locked in an approved cabinet.
- Audio files, transcribed interviews and consent forms will be stored locked away at the research institution.
- Only the signatory and the project team have access to the audio files and written material from the interview.
- You will not be recognizable in publications.

What happens to your data when we end the research project?

The project is scheduled to be completed in April 2027. Upon termination, name lists and audio files will be deleted, and personal data anonymized. Five years after the end of the project, anonymized data will be deleted.

Your rights

As long as you can be identified with the data material, you have the right to:

- access to what personal data is registered about you,
- to have personal data about you corrected,
- have personal data about you deleted,
- receive a copy of your personal data (data portability) upon request;
- to submit a complaint to the Data Protection Officer or the Data Protection Authority about the processing of your personal data.

What gives us the right to process personal data about you?

We process information about you based on your consent. On behalf of Molde University College, SIKT will assess that the processing of personal data in this project is in accordance with the data protection regulations. According to the new Personal Data Act, the data controller at Molde University College and project manager Professor Marianne Storm has an independent responsibility to ensure that the processing of your data has a legal basis. This project has a legal basis in Article 6(1a) and Article 9(2a) of the GDPR and your consent. If you have any questions about the study, or wish to exercise your rights, you are welcome to contact PhD candidate Bo Wang (bo.wang@himolde.no) or Professor Marianne Storm (marianne.storm@uis.no). Our Data Protection Officer is available via Merete Ludviksen: E-mail: merete.ludviksen@himolde.no. If you have any questions related to the assessment made by the privacy services from Sikt, you can contact us via: personverntjenester@sikt.no or phone: 73 98 40 40.

Best regards,

Marianne Storm	Bo Wang	Ingunn Pernille Mundal
Professor, PhD	Ph.D. Fellow	Professor, PhD
marianne.storm@uis.no	bo.wang@himolde.no	ingunn.p.mundal@himolde.no

Declaration of consent

I have received and understood information about the project "PeerTECH" digital peer support for people with mental illness, and am willing to participate in the study by allowing myself to be interviewed. I consent to my data being processed until the project is completed in December 2024 and have been given the opportunity to ask questions. I agree to:

Participation in the interview
That the interview is recorded with an audio recorder
That the information I contribute can be included in articles that are published

----
(Signed by project participant, date)
