# Supplementary material for: What matters in digital health technology adoption for mental healthcare? - A multistakeholder qualitative study
Source: Digit Health. 2026 Aug 10;12:20552076261469387. doi: 10.1177/20552076261469387 (PMC13458126; doi:10.1177/20552076261469387)
Supplement: Supplementary file 3 [file sj-pdf-3-dhj-10.1177_20552076261469387.pdf]

Codebook
**Code #1:** Personal adaptation<br>**Definition:** Stakeholders reflecting on the need for technology to be adapted or personalized for individual needs<br>**When to use:** Apply this code when stakeholders make comments about how technology should be user-friendly, safe and flexible to accommodate users' need and preferences.<br>**When not to use:** Do not use this if stakeholders discuss technology design in general terms without reference to individual adaptation, or when they describe adaptations at the organizational or system level rather than for individual end-users.<br>**Example:** *"The challenges are to create digital solutions that are tailored to the users' needs and the individual preferences that the users have."* (Stakeholder 23)
**Code #2**: Involving service users in the co-creation process<br>**Definition:** Stakeholders reflecting on the importance of involving service users in the co-creation and design process of digital health technology<br>**When to use:** Apply this code when stakeholders comment on the need to include service users in developing, designing, or evaluating technology, or when they discuss the value of user input in ensuring that solutions are perceived as relevant and useful. *Note* that this code can apply together with **Code #3** when commenting on both user involvement in co-creation *and* what users consider valuable.<br>**When not to use:** Do not use it when stakeholders describe service users as recipients of technology without reference to their active participation in the design or development process.<br>**Example:** *"The patient or user side should also be involved in the process, so that you get the [insight]that, they perceive the solution as something … that is better than what they've had before. Something can help them, but that it [digital solutions] can work."* (Stakeholder 26)
**Code #3:** What is valuable for service users<br>**Definition:** Stakeholders reflecting on what service users find valuable, beneficial, or meaningful in digital health technologies.<br>**When to use:** Apply this code when stakeholders comment on what matters to service users, what they perceive as helpful, or what would make a technology worthwhile from the user's perspective. *Note* that this code can be applied together with **Code #2** when commenting on both user involvement in co-creation *and* what users consider valuable.<br>**When not to use:** Do not use it when stakeholders describe the value of technology from an organizational or provider perspective.<br>**Example:** *"The patient or user side should also be involved in the process, so that you get the [insight]that, they perceive the solution as something … that is better than what they've had before. Something can help them, but that it [digital solutions] can work."* (Stakeholder 26)
**Code #4:** Peer support workers with lived experience are valuable resources<br>**Definition:** Stakeholders reflecting on the value of peer support workers in shaping technology in mental healthcare<br>**When to use:** Apply this code when stakeholders comment on peer support workers' role and contribution in service delivery, including their potential to inform the design and implementation of technology in mental healthcare<br>**When not to use:** Do not use this code when the quote is related to specific tasks or tools without reference to the value of peer support

workers lived experience.<br>**Example:** *"…we use a lot of peer support workers in our work with mental health service users, so they already have a very important role. There are many people within our municipality who have lived experience, and even more of them could be offered a type of job or positions that benefit users with lived experience than just special expertise"* (Stakeholder 24)
**Code #5:** Technology needs to be tested before implementation<br>**Definition:** Stakeholders reflecting on the importance of testing technology prior to wider implementation<br>**When to use:** Apply this code when stakeholders comment on the need to pilot, trial, or test technology on a smaller scale before broader rollout, or when they discuss how testing contributes to credibility, quality assurance, or readiness for implementation.<br>**When not to use:** Do not use it when stakeholders discuss ongoing evaluation or monitoring of technology already in routine use, or when they describe general concerns about technology quality without specifically referencing the need for pre-implementation testing.<br>**Example:** *"Testing the solutions on a small scale before expanding them is very important, so that you have great credibility when presenting it as a solution that can be important for serious mental illness."* (Stakeholder 11)
**Code #6:** Organizations need incentives for adoption<br>**Definition:** Stakeholders reflecting on how (financial and structural) incentives drive technology adoption in organizations within mental healthcare<br>**When to use:** Apply this code when stakeholders comment on the role of funding, resources, economic gains or other incentives in motivating organizations to adopt technology, or when they identify a lack of incentives as a barrier to adoption.<br>**When not to use:** Do not use this code when stakeholders discuss individual-level motivation to use technology, or when they describe organizational culture or leadership without specific reference to incentives or resource allocation as drivers of adoption.<br>**Example:** *"If there's very little reward for organizations adopting new technology, then they will not be adopted. This is an important point here - funding controls everything."* (Stakeholder 2)
**Code #7:** Leadership is vital<br>**Definition:** Stakeholders reflecting on the role of leadership driving, managing, and taking responsibility for technology adoption within organizations in mental healthcare.<br>**When to use:** Apply this code when stakeholders comment on the importance of leadership commitment, managerial oversight, or organizational accountability in the process of adopting or sustaining technology.<br>**When not to use:** Do not use this code when stakeholders discuss organizational factors such as funding, incentives, or workforce capacity without specific reference to leadership roles or managerial responsibility.<br>**Example:** *"I believe it is extremely important that when using digital solutions, they must be rooted in the sectors that can be professionally managed, and leaders who should be responsible for the management process."* (Stakeholder 13)
**Code #8:** Dedicated personnel facilitate adoption<br>**Definition:** Stakeholders reflecting on the need for designated staff with clear roles and responsibilities to facilitate the implementation and

use of technology in mental healthcare.<br>**When to use:** Apply this code when stakeholders comment on the importance of having dedicated personnel assigned to implement, manage, or operate technology, or when they discuss how clearly defined staff roles support adoption.<br>**When not to use:** Do not use this code when stakeholders discuss staffing or workforce issues in general terms without linking them to specific technology implementation responsibilities, or when they discuss leadership oversight.<br>**Example:** *"You must have dedicated staff, meaning that having someone hired to implement or use a solution. For example, if you hire psychologists to run e-health and that'll be their job, that's kind of the instruction for them"* (Stakeholder 6)
**Code #9:** Needs integrated and well-functioning digital health system<br>**Definition:** Stakeholders reflecting on the need for digital health systems that are well-integrated, interoperable, and functional<br>**When to use:** Apply this code when stakeholders comment on the need for seamless integration between digital systems, interoperability of patient information, or the consequences of working with fragmented or poorly connected infrastructure.<br>**When not to use:** Do not use this code when stakeholders discuss the usability or design of a specific technology in isolation, or when they describe general dissatisfaction with technology without reference to system integration, information flow, or infrastructure connectivity.<br>**Example:** *"There are no good system means that we constantly have to create our own systems to take care of the use in system we have, so there's no connection in patient information flow or exchange."* (Stakeholder 20)
**Code #10:** Compatible platform granting safe access to various technologies<br>**Definition:** Stakeholders reflecting on the need for a unified, secure platform that consolidates access to multiple technologies and enables safe communication across services.<br>**When to use:** Apply this code when stakeholders comment on the need for a shared or centralized platform that grants secure access to various technologies, or when they discuss problems arising from multiple disconnected platforms that fail to communicate.<br>**When not to use:** Do not use this code when stakeholders discuss system integration at the data or information flow level without reference to a unifying platform (these should be coded under **Code #9**). Also do not use when stakeholders discuss security concerns in isolation without linking them to platform consolidation or cross-service access.<br>**Example:** *"…the problem is that there are so many different platforms being established and they don't communicate well to support serious mental illness, there should be a national health platform with all technologies and agencies in there and it's secure"* (Stakeholder 24)
**Code #11:** Consistent operational support<br>**Definition:** Stakeholders reflecting on the need for ongoing technical and operational support to ensure that digital tools continue to function effectively after implementation.<br>**When to use:** Apply this code when stakeholders comment on the need for sustained technical assistance, troubleshooting, maintenance, or operational infrastructure to keep digital tools running, or when they identify a lack of such support as a barrier.<br>When not to use: Do not use this code when stakeholders discuss training or capacity-building for staff to use technology; also do not use when stakeholders discuss one-time implementation processes without reference to ongoing operational needs.

**Example:** *"There are a number of technical aspects here that make these digital tools work, so that there will be a need for a type of operational support along the way."* (Stakeholder 13)
**Code #12:** Follow European Union and national regulations<br>**Definition:** Stakeholders reflecting on the need for technology to comply with EU and national regulatory frameworks.<br>**When to use:** Apply this code when stakeholders comment on the role of EU or national regulations in shaping technology adoption, including references to regulatory alignment across countries, certification requirements, or standards for safety and treatment evaluation.<br>**When not to use:** Do not use this code when stakeholders discuss data security or privacy concerns without reference to specific regulatory frameworks; also do not use when stakeholders discuss organizational policies or internal guidelines that are not linked to formal EU or national legislation.<br>**Example:** *"Nationally, we must implement or have an endorsement of the EU regulations, so that we facilitate the use of solutions developed in Europe. That means that one agrees on a set of requirements that are the same for Norway, Sweden, France, Germany, etc. There are requirements for safety, for assessing treatment effect and side effects and so on."* (Stakeholder 9)
**Code #13:** National initiatives with resources<br>**Definition:** Stakeholders reflecting on the role of national initiatives, guidelines, and accompanying resources in supporting and legitimizing local technology adoption.<br>**When to use:** Apply this code when stakeholders comment on how national-level guidelines, strategies, or resource allocations facilitate local adoption, or when they describe using national directives to justify or drive implementation within their own organizations.<br>**When not to use:** Do not use this code when stakeholders discuss national regulations or compliance requirements (should be coded under **Code #12**). Also do not use when stakeholders discuss funding pressures without commenting to national initiatives as enabling mechanisms (should be coded under **Code #14**).<br>**Example:** *"We always look at national guidelines when we are going to develop services, because it's easier to get it introduced locally when we get some guidelines with resources saying that we should actually do it."* (Stakeholder 18)
**Code #14:** Pressure on funding structure<br>**Definition:** Stakeholders reflecting on financial constraints that hinder technology adoption in mental healthcare<br>**When to use:** Apply this code when stakeholders comment on limited budgets, cost-saving pressures, siloed funding arrangements, or structural financial barriers that constrain the ability to adopt or sustain technology.<br>**When not to use:** Do not use this code when stakeholders discuss the need for incentives to motivate adoption (should be coded under **Code #6**). Also do not use when stakeholders reference costs in passing without identifying funding structures or financial pressures as a constraining factor.<br>**Example:** *"We are under pressure and many people have to save money … The biggest barrier is finances and silo funding in municipalities."* (Stakeholder 18)
**Code #15:** Clear procurement process helps adoption

**Definition:** Stakeholders reflecting on the role of clear, accessible procurement processes in facilitating technology adoption<br>**When to use:** Apply this code when stakeholders comment on how procurement processes enable or hinder adoption, including references to national approval mechanisms, standardized purchasing pathways, or the challenges smaller municipalities face in navigating procurement independently.<br>**When not to use:** Do not use this code when stakeholders discuss funding or financial barriers without specific reference to procurement processes (which should be coded under **Code #14**). Also do not use it when stakeholders discuss regulatory approval in terms of safety or compliance standards rather than purchasing and dissemination pathways (which should be coded under **Code #12**).<br>**Example:** *"I think that perhaps national authorities need to make some procurements, possibly approve technologies, so that it becomes easier for municipalities and specialist health services to get started and use the tools that you are developing. Because once you have developed it, it is not automatic that it will spread like wildfire. Many municipalities are small, they do not have knowledge about procurement."* (Stakeholder 9)

From codes to themes	How themes and NASSS domains connect and explain each other
**Code #1-4** addressed the user-facing dimension of technology adoption, including how technology should be personalized, co-created with service users, and grounded in the experiential knowledge of peer support workers. These codes formed **Theme 1: Personalized adaptive digital health technology through co-creation and peer support**	Theme 1 aligns with the demand-side *value proposition* in the NASSS framework, as it addresses whether the technology is perceived as worthwhile by the people it is intended to serve. The *value proposition* concerns whether a technology offers a credible and desirable improvement over existing care from the perspective of end-users, which corresponds to the theme's emphasis on personalization, co-creation, and grounding technology in the experiential knowledge of peer support workers
**Codes #6-8** addressed the organizational conditions needed to drive adoption, including incentives, leadership, and dedicated personnel. These codes formed **Theme 2: Evidence-informed organizational adoption for digital mental healthcare**	Theme 2 aligns with the supply-side *value proposition* in the NASSS framework, as it addresses whether technology adoption is viable and justifiable from the organization's perspective. The *value proposition* also concerns whether the organizational commitment and resources for adoption is sustainable, which resonates to the emphasis of this theme for technology adoption.
**Code #9-11** discussed the technical infrastructure required to sustain adoption with important considerations of system integration, platform unification, and ongoing operational support all come together. These codes formed **Theme 3: A well-integrated and**	Theme 3 aligns with the NASSS domain of *organization*, as it addresses the technical infrastructure and operational capacity that organizations must have in place to sustain technology adoption. This domain concerns whether an organization has the readiness, resources,

**sustainable digital health system to support mental healthcare**	and internal systems to support implementation, which corresponds to the theme's emphasis on system integration, a unified secure platform, and consistent operational support as organizational prerequisites for sustainability of technology.
**Code #12-15** addressed how macro-level regulatory and strategic environment (i.e., EU and national regulations, national initiatives, funding structures, and procurement pathways) collectively safeguard technology adoption. These codes formed **Theme 4: Clear and enforced national regulatory frameworks and strategies**	Theme 4 aligns with the NASSS domain *wider system*, as it centered around the macro-level policy and regulations shaped technology adoption. The *wider system* domain concerns how external forces enable or constrain adoption at scale. This corresponds to the theme's emphasis on EU and national regulatory compliance, the enabling role of national initiatives with accompanying resources, funding pressures within municipalities, and the need for clear procurement pathways to facilitate dissemination beyond individual organizations.
